# Influence of Powder Type and Layer-Dependent Energy Input on Multi-Layer Laser Cladding of Ductile Cast Iron

**DOI:** 10.3390/mi17080877

**Published:** 2026-07-23

**Authors:** Meryem Altay, Hakan Aydın, Adem Karşı

**Affiliations:** 1 Engineering Faculty, Mechanical Engineering Department, Bursa Uludag University, 16059 Bursa, Turkey; 2 Coşkunöz CMF R&D Center, Bursa Organized Industrial Zone, Sarı Street No:7, 16215 Bursa, Turkey

**Keywords:** laser cladding, multi-layer deposition, layer dependent energy input, powder material, macrostructure, residual stress, thermal history

## Abstract

This study investigates the effects of powder type and layer-dependent energy input on the structural and mechanical performance of multi-layer laser cladding applied on FGS600-3A ductile cast iron. Three cladding powders (Ferro 55, Castolin 16604, and Metco 41C) were deposited under constant and variable energy input to examine the effects on macrostructure, porosity, microhardness, residual stresses, and thermal history. The results demonstrate that powder composition plays a decisive role in deposition quality. Ferro 55 exhibited the lowest porosity and the most favorable hardness distribution, whereas Metco 41C had high porosity, low hardness, and severe transverse cracking. Castolin 16604 displayed intermediate performance with deeper high-hardness penetration. Layer-dependent energy strategies improved porosity and hardness behavior for Ferro 55 and Castolin 16604; however, excessively low energy input limited hardness depth. Residual stress measurements revealed low stress levels for Ferro 55, compressive stress for Castolin 16604, and high tensile stress for Metco 41C, correlating strongly with cracking tendency. Overall, Ferro 55 and Castolin 16604 were identified as suitable candidates for multi-layer repair and surface modification of cast iron molds, while Metco 41C demonstrated limited applicability due to its porosity, hardness, and stress characteristics. This study emphasizes the importance of layer-specific parameter optimization to achieve defect-free and mechanically strong laser cladding.

## 1. Introduction

Laser cladding is a surface modification and repair technique, widely applied in the automotive and molding industries. Powder material is transferred into a localized melt pool generated by a high-energy laser beam, resulting in metallurgical bonding. Laser cladding has been extensively investigated by numerous researchers [1,2,3,4,5,6,7]. Compared to conventional surface engineering and repair methods, laser cladding offers several advantages, including precise control of heat input, low porosity, narrow heat-affected zones, and strong bonding between the clad layer and substrate. These features minimize distortion and cracking. However, the need for precise parameter control remains a limitation of this method. Particularly in the molding industry, laser cladding has increased in importance as a repair method for worn or damaged molds, where complete replacement is often expensive and time-consuming. Beyond repair, it is also used to enhance component service life.

Numerous studies have examined how processing parameters affect the microstructural and mechanical performance of laser coatings. Parameters such as laser power and scanning speed strongly influence layer geometry, porosity, and bonding quality. Altay et al. reported that changing heat input during martensitic stainless steel cladding significantly alters porosity, residual stress, microhardness, and thermal history [8]. Qian et al. investigated multi-layer Ni-based claddings on 316L steel with gradually increasing laser power (1.8–2.4 kW). As energy input increased, grain coarsening occurred, and hardness decreased, though wear resistance improved. The coatings had nearly three times the substrate hardness, highlighting the importance of layer-specific heat input for optimizing hardness–wear behavior [9]. A layer-dependent analytical model was later proposed to predict optimal parameters for depositing 07Cr15Ni5 steel on a CuCr substrate via directed energy deposition. Considering variations in thermophysical properties and absorption with layer number, the model produces printability maps closely matching experiments [10]. Yu et al. simulated laser cladding repair of cracked gears using ANSYS. Optimal parameters (140 W, 8 mm/s) yielded a defect-free layer with 1.22 times higher tensile strength and 2.05 times higher hardness than the substrate [11]. Tang et al. combined numerical and experimental approaches for multi-layer and multi-pass cladding of 6061 Al alloy. Increasing cladding passes raised substrate temperature, improved bonding, and reduced residual stresses, though excessive passes lowered microhardness and introduced porosity near overlap regions [12]. Recent studies emphasize layer-dependent energy input strategies, showing that controlled variation in specific energy between layers can refine microstructure and mechanical properties. However, further systematic research is still needed to establish strong correlations between process parameters, inter-layer energy distribution, and resulting performance.

This study systematically investigates multi-layer laser cladding using three commercially available mold-repair powders (Ferro 55, Castolin 16604, and Metco 41C) and layer-dependent energy input strategies to fill the existing research gap. Unlike previous studies focusing on a single powder material under constant process parameters, this study systematically compares different powders under identical processing conditions and evaluates layer-dependent energy input for different alloy systems. The novelty of this work lies in the systematic investigation of multi-layer laser cladding on FGS600-3A ductile cast iron, a widely used mold material, through the comparison of three commercially available powders and the implementation of a layer-dependent energy input strategy. The combined evaluation of macrostructure, porosity, microhardness, residual stress, and thermal history provides a comprehensive understanding of their interactive effects on coating performance. The findings provide guidance for powder material selection and process optimization in industrial mold repair.

## 2. Experimental Details

The laser cladding method was employed to deposit Ferro 55, Metco 41C and Castolin 16604 powder material onto FGS600-3A ductile cast iron, used in sheet metal forming molds. The laser cladding process was performed on FGS600-3A mold specimens with dimensions of 120 × 30 mm. The substrate was cast by our group to simulate sheet-metal-forming molds. Before laser cladding, a 1 mm deep groove was machined into the mold surface using CNC milling to provide a smooth cladding area. The chemical compositions of these materials are shown in Table 1.

The Ferro 55 powder used in this study is compositionally comparable to AISI 431 martensitic stainless steel. Materials in this class typically have a tensile strength in the range of 850–1000 MPa, a yield strength of about 665 MPa, and an elongation of approximately 12%. The hardness is generally between 248 and 302 HB. The particle size distribution of Ferro 55 powder lies between 45 and 125 µm. The substrate material, FGS600-3A ductile cast iron, exhibits a tensile strength of nearly 600 MPa, a yield strength of about 370 MPa, and roughly 3% elongation, with hardness values commonly reported between 170 and 270 HB. Metco 41C powder corresponds to an alloy system based on 316L austenitic stainless steel with Brinell hardness 149 B, Rockwell B hardness 80 HRB, Vickers hardness 155 HV, ultimate tensile strength 515 MPa, yield tensile strength 205 MPa, elongation 60%, elasticity modulus 193 GPa, and powder size 106 + 45 µm. EuTroloy Castolin 16604 produces a coating that undergoes significant work hardening and develops a very fine martensitic microstructure. This allows the deposited layer to withstand elevated temperatures, rapid thermal fluctuations, and corrosive environments, while exhibiting strong resistance to cracking. Owing to these properties, the material is commonly employed in hot and cold metal-forming applications.

The powders employed in this study (Ferro 55, EuTroloy Castolin 16604, and Metco 41C) were commercially available industrial laser cladding powders and were used in as-received condition without additional drying or conditioning. The manufacturers provided the nominal chemical composition and particle size range; however, detailed information regarding powder morphology, particle size distribution, flowability, apparent density, oxygen content, and powder conditioning history was not available to the authors. Since the objective of this study was to compare the performance of commercially available powders under identical laser cladding conditions and different layer-dependent energy input strategies, rather than to investigate the influence of feedstock powder characteristics, all powders were processed using the same laser cladding system, powder feeder, shielding gas conditions, and processing parameters. Therefore, the comparative assessment presented in this study remains internally consistent, and the absence of these proprietary powder characteristics does not compromise the validity of the comparative conclusions. This limitation has been acknowledged and will be addressed in future studies through comprehensive powder characterization.

The laser cladding experiments were performed using a KUKA KR 90 R3100 Extra six-axis robotic system (KUKA Deutschland GmbH, Augsburg, Germany) integrated with a KUKA KL 1500-3T linear stage, both operated through Toplas 3D V3 control software. The cladding setup employed a LASERLINE LDF 4000-100 diode laser (Laserline GmbH, Mülheim-Kärlich, Germany), capable of delivering up to 4000 W of power within a wavelength range of 900–1070 nm. The laser beam diameter was 3 mm with a beam quality of 30 mm·rad. The minimum spot size was 450 µm at a working distance of 150 mm, but this condition was not employed in this study. Laser energy was transmitted through a 30 m optical fiber with a 600 µm core diameter and a numerical aperture of 0.2. Process cooling was provided by a DELTATHERM LTK 1–4 nozzle-cooling system (DELTATHERM Hirmer GmbH, Much, Germany), and powder was delivered using an ERLAS GmbH cladding nozzle (ERLAS Erlanger Lasertechnik GmbH, Erlangen, Germany). Argon shielding gas was supplied at 5 L/min, maintaining a constant 12 mm nozzle–substrate standoff distance. Powder feeding was controlled by an Oerlikon Twin-120A system (Oerlikon Metco AG, Wohlen, Switzerland), operating at a feed rate of 13.6 g/min, a rotation speed of 3 rpm, a cooling water flow of 1.8 L/min, and a water pressure between 0.22 and 0.3 MPa.

The base material used in this study was FGS600-3A. Several samples were produced using different powder feedstock materials and process parameters. For comparison purposes, Samples 1, 2, and 3 were fabricated using Ferro 55, Metco 41C, and Castolin 16604 powders, respectively, under identical process conditions (power = 1.3 kW; scanning speed = 8 mm/s; Hatch spacing = 1.05 mm). The used process parameters are shown in Table 2. The hatch spacing parameters were kept constant at 1.05 mm, which corresponds to 65% overlap rate; it is based on our previous studies [13].

**Figure 1:**
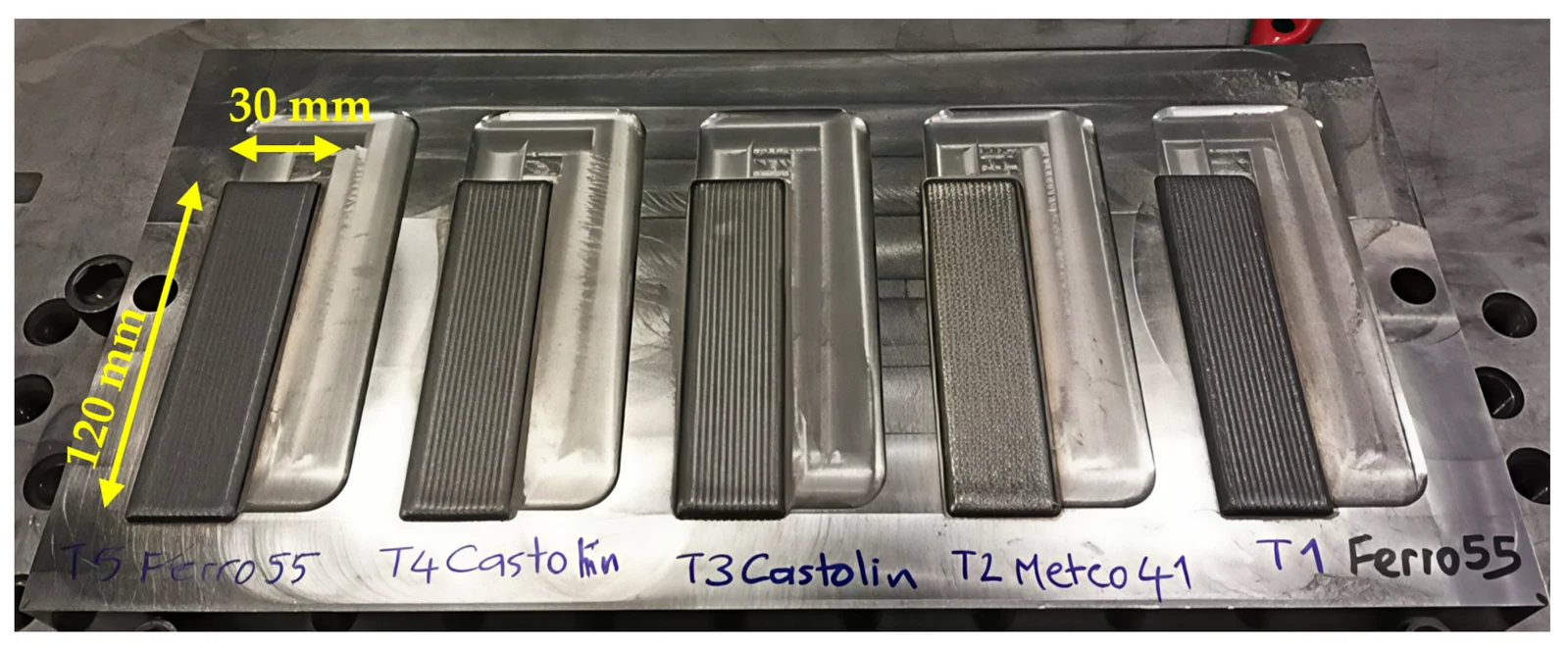
Produced mold specimens for all materials.

To investigate the influence of energy input, multi-layer deposition strategies were applied for Samples 4 and 5. The energy input was calculated using Equation (1) in Table 2, which relates it to laser power, scanning speed, beam diameter, and overlap distance; the heat input values are presented in Table 2 [13]. In these cases, Castolin 16604 (Sample 4) and Ferro 55 (Sample 5) powders were deposited in three successive layers, where the first and third layers were produced with lower energy input (Power = 1.1 kW; Scanning speed = 12 mm/s; hatch spacing = 1.05 mm), while the second layer was deposited with higher energy input (Power = 1.3 kW; Scanning speed = 8 mm/s; Hatch spacing = 1.05 mm). Based on the preliminary evaluation according to constant energy conditions (Sample 1, 2, 3), vertical cracking was observed in Sample 2. Therefore, Metco 41C was not included in the layer-dependent variable energy input experiments; Ferro 55 and Castolin 16604 were selected for further investigation. The used process parameters are shown in Table 2. The produced samples, along with the base material, are shown in Figure 1.

(1) $$Energy\;Input=\frac{Laser\;Power}{Scanning\;Speed*Beam\;Diameter*Hatch\;Spacing}$$

For the microstructural examinations, samples were cut perpendicular to the cladded layers using electrical discharge machining (EDM). The prepared cross-sections were subsequently ground with waterproof SiC papers ranging from 180 to 1200 grit and then polished using 1 µm and 0.3 µm alumina suspensions on a FORCIPOL 2V polishing system (Metkon Instruments Inc., Bursa, Türkiye). Etching was carried out with Kalling-1 reagent (1.5 g CuCl_2_, 33 mL HCl, 33 mL ethanol, and 33 mL distilled water), applied manually for 2–10 s. Microstructural observations at different magnifications were performed using a NIKON ECLIPSE MA100 inverted metallurgical microscope equipped (Nikon Corporation, Tokyo, Japan) with CLEMEX imaging software (version 6.0.026). Porosity evaluation was conducted through digital image processing using the NIS Elements-D (version 5.42.x) platform. The optical images were converted to grayscale and contrast-enhanced to improve pore distinction, after which individual pores were manually segmented from the matrix. Software was then used to compute pore areas and total image area, allowing porosity to be reported as an area percentage. Microhardness measurements were taken on both transverse and longitudinal cross-sections with a DUROLINE-M Vickers microhardness tester (Metkon Instruments Inc., Bursa, Türkiye), applying a 50 g load for 10 s at depth intervals of 100 µm from the coating surface to the substrate. Microhardness indentations were performed starting from 100 µm below the coating and extending through the interface into the base metal up to approximately 11,000 µm. A total of 22 representative measurement points are presented in the microhardness profiles. Residual stresses in the cladded layers were determined using X-ray diffraction (XRD), a non-destructive method capable of assessing stresses over relatively large surface areas without physical contact. Prior to analysis, all specimens were electropolished. Measurements were performed with an A-STRESSTECH diffractometer (Stresstech Oy, Vaajakoski, Finland) operating at 30 kV, with a ±45° tilt angle and an oscillation of ±0.003 mm. A non-destructive sin^2^ψ method was employed using an X-ray diffractometer. Cr-Kα radiation was used as the X-ray source. Measurements were recorded at ψ tilts of 0°, 45°, and 90° at eight predefined locations on each specimen. The diffraction peak was recorded within a 2θ range of approximately 156–165°, with an angular step size of 0.1° and an exposure time of 5 s per step. A collimator with a 2 mm spot diameter was used to define the irradiated area.

The laser cladding operations were monitored using an OPTRIS PI450i infrared camera (Optris GmbH, Berlin, Germany), while real-time thermal data were analyzed experimentally through OPTRIS PIX Connect software. The camera was capable of measuring temperatures between 625 and 1900 °C, with a pixel resolution of 764 × 480 and a measurement frequency of 80 Hz. An emissivity value of 0.95 was used for temperature calculations. The camera was factory-calibrated prior to delivery, and no additional calibration was performed before the experiments. Temperature evaluation was carried out within a predefined rectangular region of interest (ROI) covering the laser cladding area. To minimize interference from the powder plume, molten spatter, and reflected laser radiation, the infrared camera was positioned at an oblique side angle with respect to the laser beam, allowing continuous monitoring of the cladding process. The substrate had dimensions of 120 × 30 mm.

## 3. Results and Discussion

### 3.1. Macrostructure and Porosity Analysis

**Figure 2:**
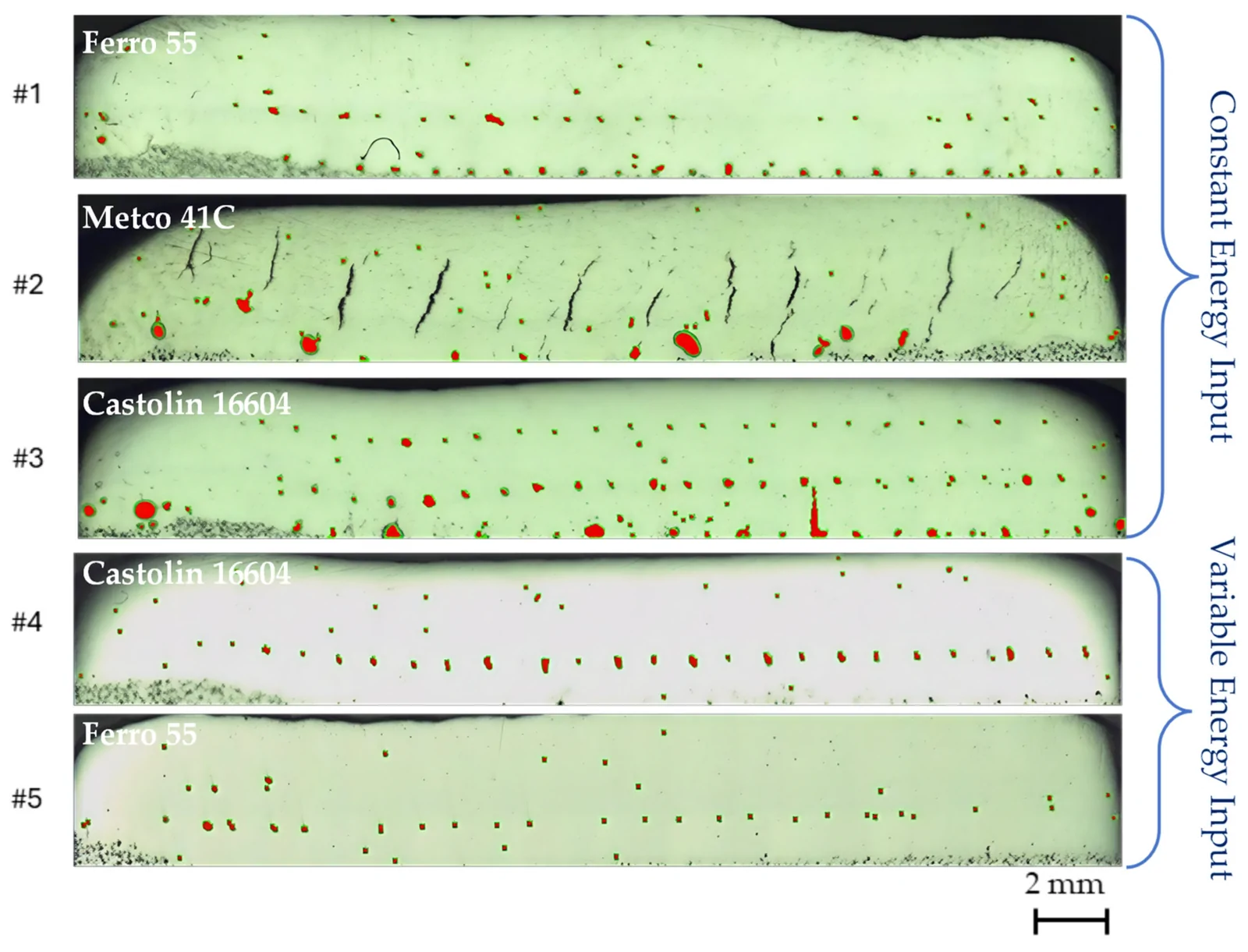
Digital image processing results of transverse cross-sectional surfaces for all specimens.

**Figure 3:**
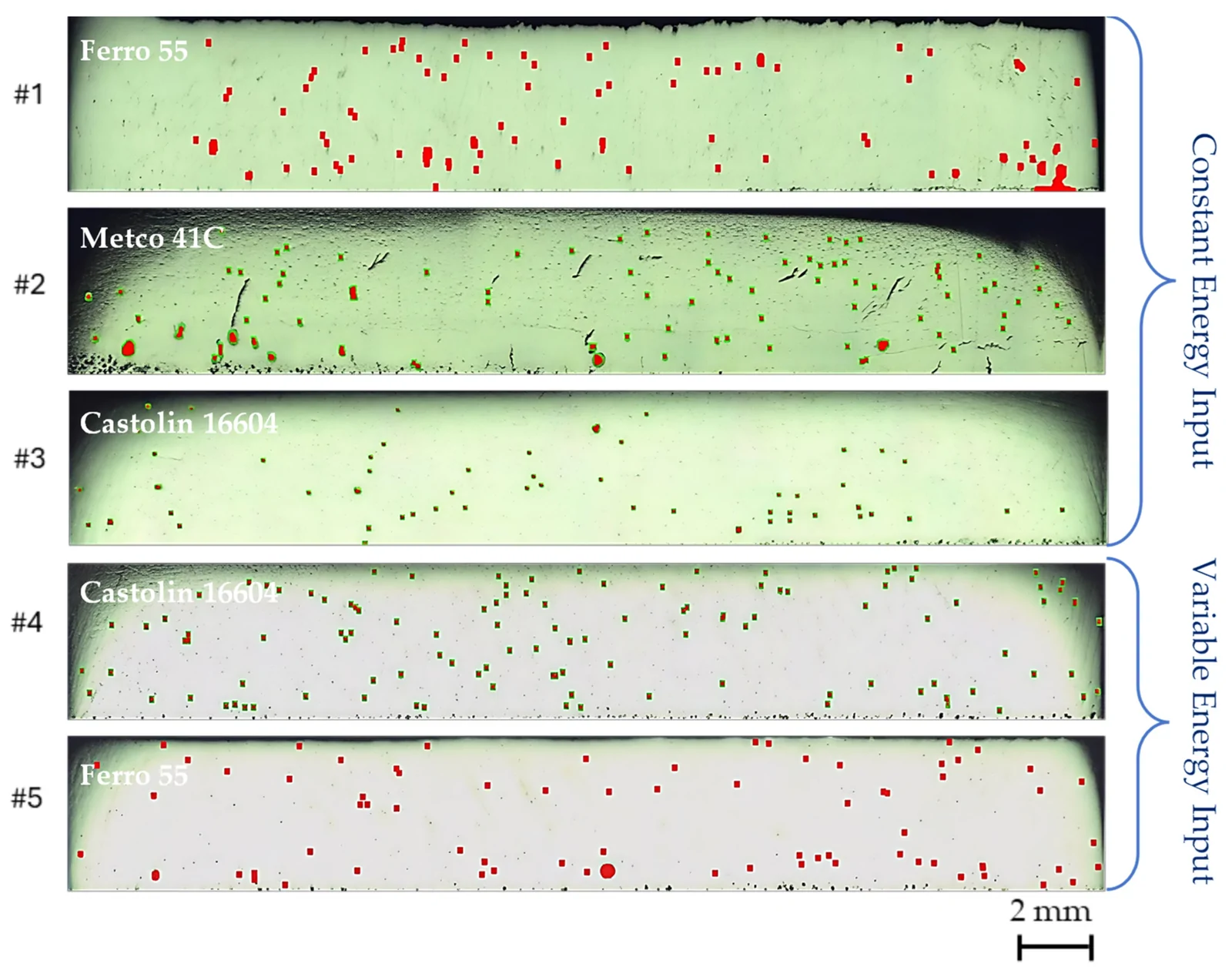
Digital image processing results of longitudinal cross-sectional surfaces for all specimens.

**Figure 4:**
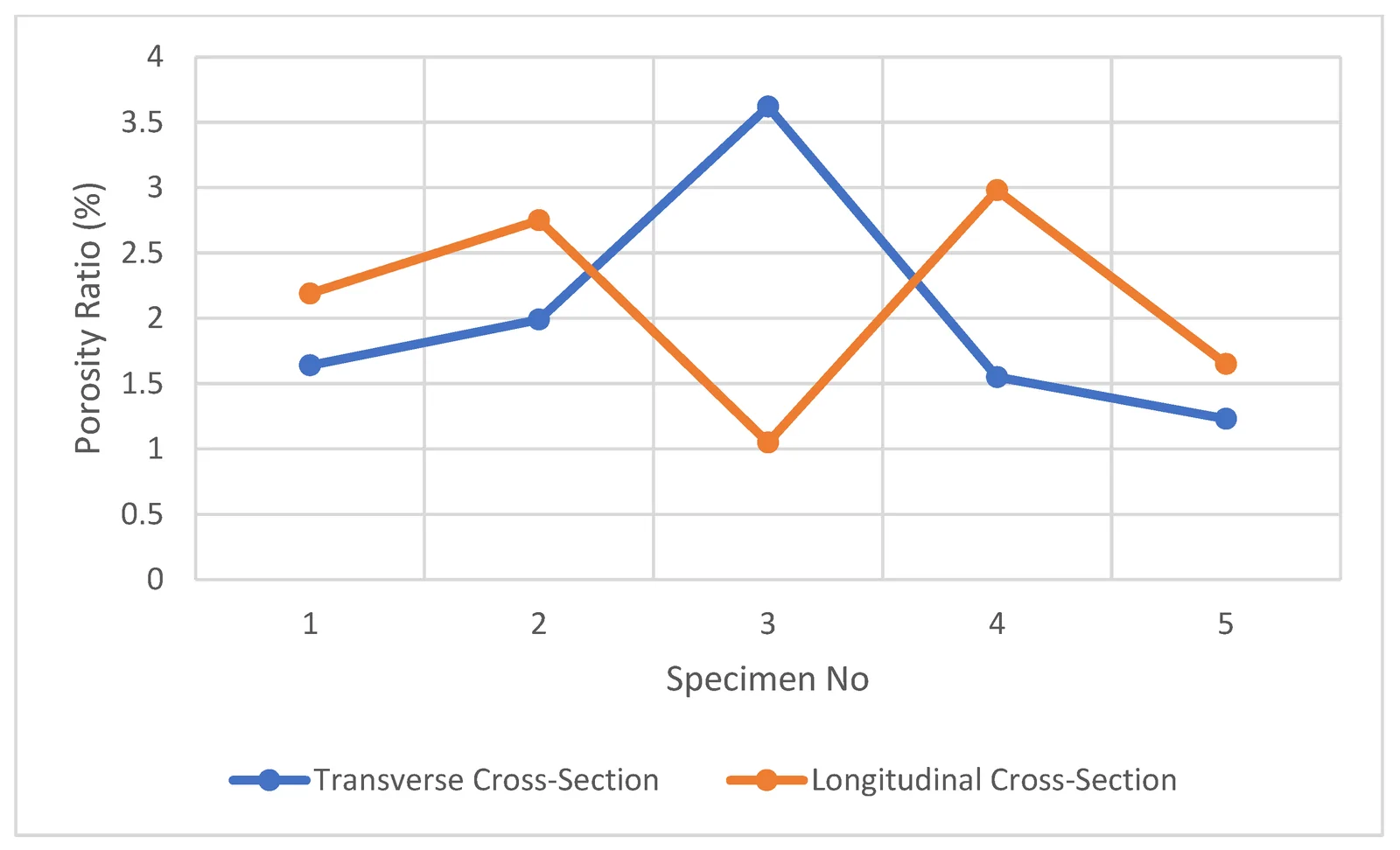
Pore fraction of cross-sectional surfaces.

The porosity images obtained through digital image processing are shown in Figure 2 and Figure 3 for the cross-sectional surfaces. The total area and pore area were calculated to determine the porosity ratio, and the corresponding results are summarized in Table 3. When the process parameters of laser power = 1.3 kW, scanning speed = 8 mm/s, and hatch spacing = 1.05 mm were employed, thus providing identical energy input to all layers for the three cladding powders, the lowest porosity levels in the transverse sections were obtained in the order of Ferro 55, Castolin 16604, Metco 41C,(Figure 4). Ferro 55 powder material exhibited the lowest porosity in transverse sections, whereas the highest porosity was observed in Castolin 16604 powder material with constant parameters. In addition, vertical cracks were clearly evident in the transverse section of Metco 41C. In the longitudinal section, Metco 41C exhibited a relatively high porosity fraction, whereas Castolin 16604 had a low porosity level under constant processing parameters. Because the Metco 41C cladding exhibited cracks that occurred together with the highest transverse porosity, it was excluded from the variable energy experiments. Therefore, the effect of layer-dependent energy input was not evaluated for Metco 41C.

The results show that crack formation was observed predominantly in the Metco 41C coating, where the cracks propagated mainly along the deposited layers in the build direction toward the top of the clad layer. Crack formation generally concentrated in the central region of the clad layer. The crack length density was calculated by dividing the total crack length by the total analyzed area. A total of 16 cracks were identified in the transverse section, whereas 12 cracks were identified in the longitudinal section. The Metco 41C coating exhibited a crack length density of 0.176 mm^−1^ in the transverse section and 0.117 mm^−1^ in the longitudinal section, indicating that crack propagation was more pronounced in the transverse cross-section.

The clad geometry parameters obtained from the cross-sectional analyses are summarized in Table 3. The dilution rate was determined using the following relationship: the penetration depth divided by the sum of the coating height and penetration depth, expressed as a percentage. The measured penetration depth varied between 400 and 700 μm, corresponding to dilution rates ranging from 8.81% to 22.43%. Among all specimens, the Metco 41C coating exhibited the highest dilution rates, reaching 15.05% and 22.43% in the transverse and longitudinal sections, respectively. This may have contributed to the higher porosity, crack formation, and tensile residual stresses observed in the Metco 41C coating. In contrast, the Ferro 55 and Castolin 16604 coatings generally exhibited lower dilution rates, which are expected to reduce substrate material transfer into the coating. Furthermore, all specimens exhibited good metallurgical bonding at the coating/substrate interface.

When variable energy inputs were interpreted together for Ferro 55 and Castolin 16604 powders, Ferro 55 had a lower porosity ratio. A similar trend was also observed in the constant energy input. In addition, for Castolin 16604, the longitudinal porosity increased from 1.05% to 2.98% after applying variable energy input. Overall, the application of layer-dependent energy input to Ferro 55 resulted in a reduction in porosity, decreasing from 1.64% to 1.23% in the transverse surface and from 2.19% to 1.65% in the longitudinal surface.

In this study, the porosity analysis was primarily based on quantitatively determining the pore fraction. Although a detailed microscopic characterization was not conducted to explicitly classify pore types, the observed pore morphologies provide us with the possible defect formation mechanisms. The spherical pores are commonly associated with gas entrapment, whereas irregular pores are typically attributed to lack of fusion defects, emerging from insufficient melting and incomplete bonding between adjacent tracks [14,15,16]. A detailed pore classification in laser cladding was addressed in our previous work [17]. The Metco 41C coating exhibited cracks propagating through the coating thickness toward the surface. Crack formation indicates a higher susceptibility to cracking compared with the other powders. The lower porosity obtained with Ferro 55 and Castolin 16604 suggests more stable cladding conditions.

### 3.2. Microhardness

The Metco 41C powder exhibited hardness values at the surface that were lower than those of the cast substrate material, demonstrating substantially lower hardness compared with the other powders. A slight increase in hardness was observed near the substrate; however, these values remained significantly lower than those of the other powders. A similar trend of the hardness curve was observed by Telesang et al. [18]. When specimens produced with different powders under identical energy input (Sample 1 and Sample 3) were compared, the hardness values near the surface appeared to be similar. In contrast, the Ferro 55 and Castolin 16604 powders were able to maintain high hardness values at greater depths.

**Figure 5:**
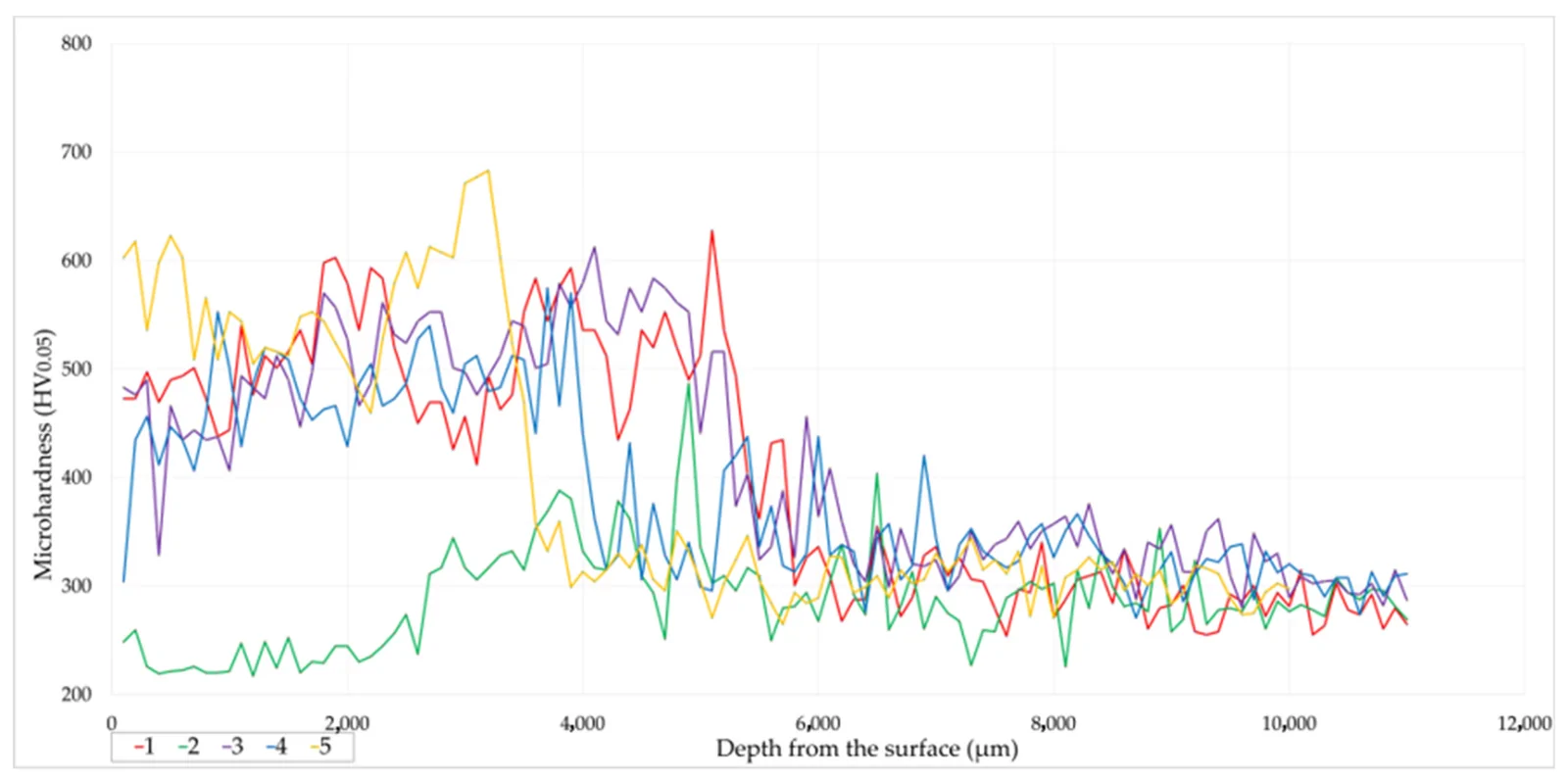
Measured microhardness values of transverse surface.

When varying energy inputs were applied between layers for the Ferro 55 and Castolin 16604 powders, the Castolin powder showed a reduction in hardness, and the high-hardness region did not extend to deeper layers. For the Ferro 55 powder, higher hardness values were achieved near the surface, although the depth at which these high values were sustained decreased. Overall, applying different energy inputs across the layers led to a reduction in the depth of the region exhibiting elevated hardness (Figure 5). Higher cooling rates associated with lower energy input promote increased hardness, whereas excessive heat input tends to soften the coating [19]. Additionally, repeated thermal cycling in multi-layer coatings reduces the depth of hardened regions due to reheating of underlying layers [20]. Accordingly, the lower hardness of Metco 41C should be interpreted as a consequence of its austenitic alloy design rather than as an indication of poor coating quality. Nevertheless, for applications where wear resistance is the primary service requirement, the higher hardness obtained with Ferro 55 and Castolin 16604 is expected to be more advantageous.

According to the hardness measurements in the longitudinal sections, the high-hardness regions did not extend to greater depths. The Metco 41C powder again exhibited surface hardness values lower than those of the cast substrate, showing substantially lower hardness compared with the other powders. However, a significant increase in hardness was detected near the substrate. This increase may be attributed to carbon diffusion from the substrate to the melt pool of the first layer. When comparing the specimens produced with different powders under identical energy input (Samples 1 and 3), the highest hardness values were observed in the Ferro 55 powder, while the lowest were obtained with the Metco 41C powder. Notably, the Castolin 16604 powder enabled the high-hardness region to extend deeper into the material, whereas the Metco 41C powder did not exhibit such depth penetration of elevated hardness.

**Figure 6:**
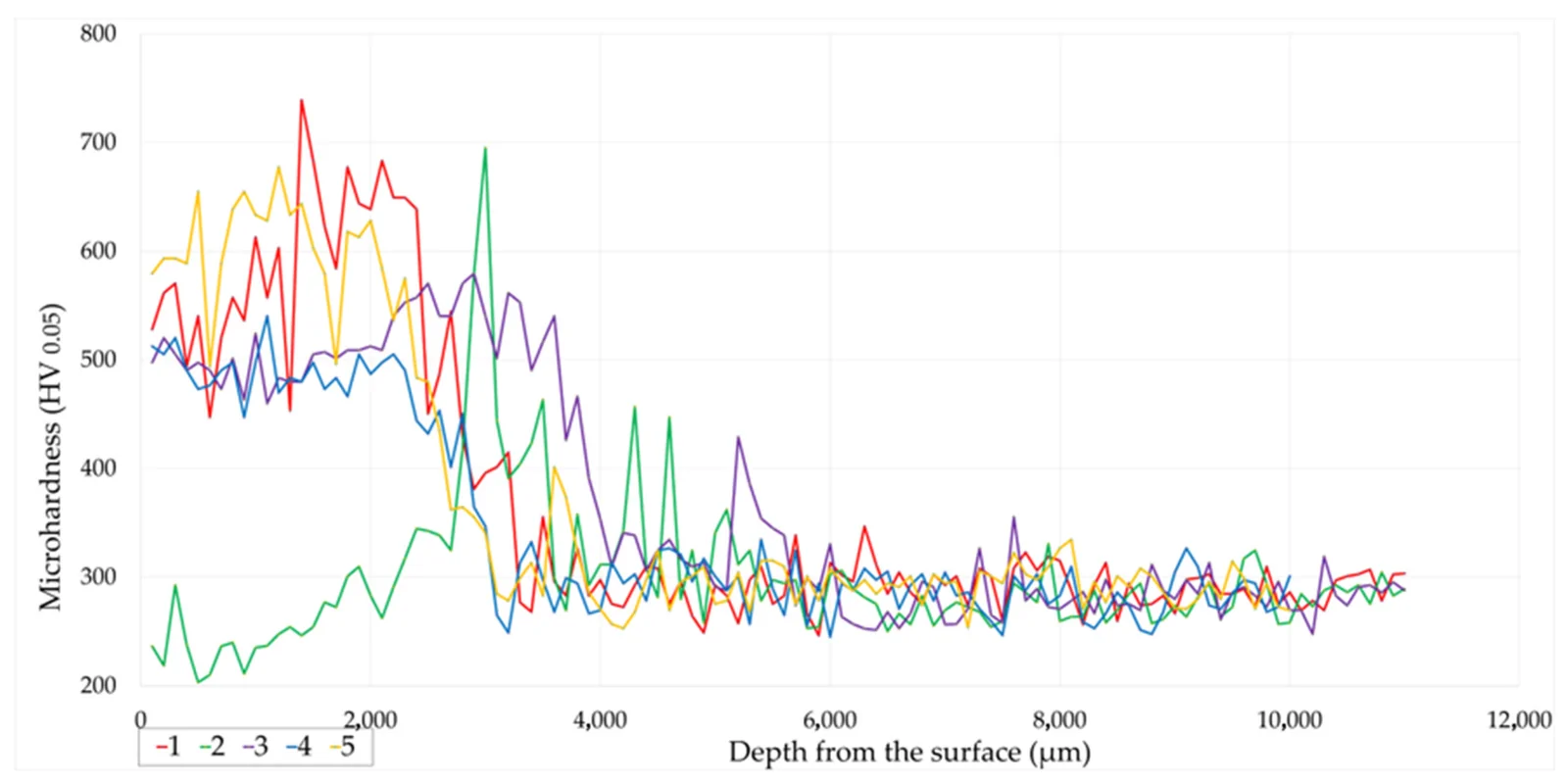
Measured microhardness values of longitudinal surface.

When varying energy inputs were applied between layers for the Ferro 55 and Castolin 16604 powders, the Castolin powder exhibited reduced hardness, and the depth of the high-hardness region diminished. In the Ferro 55 powder, elevated hardness values were observed near the surface; however, the depth at which these high values were sustained became shallower. Similarly, in the longitudinal section, applying different energy inputs across layers decreased the depth of the region exhibiting elevated hardness (Figure 6).

In laser cladding processes, high cooling rates typically promote the formation of non-equilibrium microstructures such as martensitic or fine dendritic structures, which cause differences in hardness. Ferro 55 and Castolin 16604 are more likely to undergo martensitic or carbide-stabilized transformations due to their alloying elements, resulting in higher hardness. On the other hand, Metco 41C may exhibit a relatively softer microstructure, which explains its lower hardness values near the surface.

### 3.3. Residual Stress

Crack formation in laser-cladded ductile cast iron was primarily governed by the formation of brittle structures at the interface due to rapid solidification and carbon enrichment. In addition, the diffusion of carbon from the substrate into the molten pool promotes the formation of hard and brittle phases, increasing crack susceptibility in the coating–substrate interface. Unlike conventional studies focusing only on process parameters, this work considers the residual stress in determining crack susceptibility for different alloy powders.

Table 4 presents the residual stress measurement results obtained by XRD for the 0°, 45°, and 90° orientations. The analysis of the stresses along the welding direction indicates that the Castolin powder deposit developed notable compressive residual stresses, whereas the Ferro 55 powder resulted in only relatively low compressive stress. In contrast, the Metco 41C powder exhibited predominant tensile residual stress. Despite these stress states, no visible cracks were detected in the longitudinal sections of the specimens, indicating that the residual stresses generated along the welding direction were insufficient to cause deformation or cracking (maximum compressive stress: 261 MPa). In the direction perpendicular to the welding path, compressive residual stresses were observed for the Ferro 55 powder, whereas tensile residual stresses emerged in the Metco 41C and Castolin powder deposits. Particularly in the Metco 41C specimen, the tensile residual stress reached considerably high levels (~400 MPa). The pronounced cracking observed in the transverse section of this specimen can be reasonably attributed to these elevated tensile stresses. The residual stresses measured at 45° relative to the welding direction also showed a similar trend: the Metco 41C specimen, where severe cracking was observed, exhibited dominant and high tensile residual stresses (~460 MPa). The higher residual stress observed in the 45° orientation for the Metco 41C specimen can be attributed to the anisotropic nature of laser cladding. In particular, the laser scanning direction induces thermal gradients along the 0° direction, while the transverse direction (90°) is primarily affected by lateral heat transfer. The 45° orientation represents a superposition of these principal stress components. It caused the combined accumulated effect of both longitudinal and transverse stresses. In contrast, specimens produced with Ferro 55 and Castolin powders showed low-level compressive residual stresses in this orientation.

When the maximum principal residual stresses of the specimens were examined, tensile stresses dominated in all cases. The specimen produced with Metco 41C exhibited very high tensile residual stresses (up to 490 MPa), whereas the Castolin powder produced moderate tensile stresses. The Ferro 55 specimen, on the other hand, exhibited only very low tensile residual stresses (~3 MPa). The minimum principal residual stresses showed that tensile stresses remained dominant in the Metco 41C specimen, while compressive stresses prevailed in the Ferro 55 and Castolin 16604 specimens, with a higher compressive magnitude. Similar findings have been reported in the literature, where multi-layer laser cladding often generates tensile residual stresses in upper layers due to the constrained cooling, while compressive stresses are more common in lower, reheated regions [21]. It is known that increased heat input promotes higher tensile residual stresses, whereas lower or controlled heat input can help maintain compressive stress states [22].

The Metco 41C specimen exhibited significantly higher tensile residual stresses than the Ferro 55 and Castolin 16604 specimens. This behavior can be further interpreted in terms of solidification characteristics and thermal contraction mismatch. Rapid solidification promotes the formation of a constrained-shrinkage zone during cooling [23,24].

### 3.4. Thermal Assessment

Thawari et al. conducted a study to monitor the thermal history during multi-layer laser coating of Stellite 6 [22]. They said that thermal gradients and rapid heating and cooling lead to distortion in laser cladding. Therefore, process parameters need to be optimized in multi-layer coatings. Thermal analyses have been conducted for various materials, considering both single-track [25,26] and multi-layer configurations [27]. Accordingly, in order to address the need for further investigation of Metco 41, Ferro 55, Castolin 16604 materials, their thermal history has been discussed. This study focused on the analysis of time and temperature data obtained from experimental measurements.

**Figure 7:**
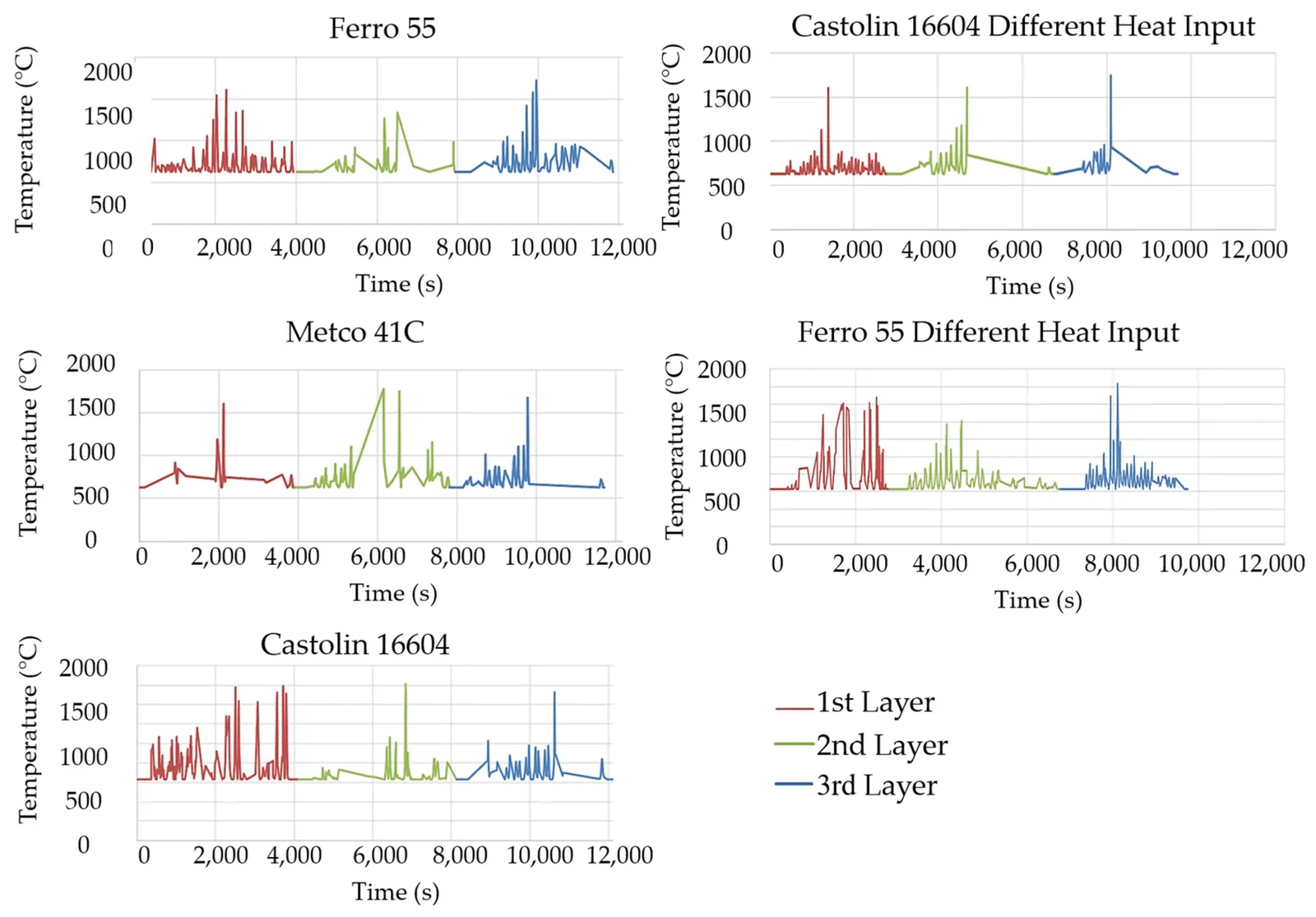
Time–temperature graphics for all specimens.

Time-dependent thermal profiles for all specimens are presented in Figure 7. For the Ferro 55 sample, the maximum temperature in the third layer was noticeably higher, whereas in the second layer, the peak temperature did not exceed 1500 °C. In both the second and third layers, elevated temperatures spread over a relatively larger region, which could be attributed to the heat input retained from the first-layer deposition. For the Metco 41C specimen, the maximum temperature in all layers exceeded 1500 °C. More pronounced spattering was observed in the first layer. Overall, no significant differences in maximum temperature or heated area were observed between layers. For Castolin 16604 (Sample 3), the maximum temperature in all layers was approximately 1600 °C; however, the extent of the heated region increased in the upper layers. In the Castolin 16604 specimen with varying energy input (Sample 4), although lower heat input was applied to the first and final layers, the maximum temperature still remained around 1600 °C across all layers. Similar to the constant energy case, the heated zone became broader in the upper layers. The Ferro 55 specimen with varying energy input (Sample 5) demonstrated a thermal distribution comparable to that of the constant energy Ferro 55 specimen (Sample 1). The maximum temperature again occurred in the third layer. Despite the second layer receiving higher heat input relative to the others, its peak temperature did not surpass 1500 °C. This behavior appeared to be associated with the chemical composition and physical properties, particularly the thermal conductivity, of the Ferro 55 powder. Similar to the constant energy condition, elevated temperatures in the second and third layers extended over a wider region due to the heat contributed by the first layer.

The peak temperatures determined from the thermal history data for each deposited layer are presented in Table 5. The extracted data show that Samples 1, 4, and 5 reached their highest peak temperatures during the deposition of the third layer (1750, 1762, and 1850 °C, respectively), indicating heat accumulation as successive layers were deposited. In contrast, Sample 2 exhibited the highest peak temperature in the second layer (1758 °C), while Sample 3 showed relatively similar peak temperatures for all three layers. These results demonstrate that both powder type and layer-dependent energy input significantly influence the thermal history during the laser cladding process. The higher peak temperatures observed in the upper layers are attributed to thermal accumulation. Increased heat accumulation affected the porosity rate and residual stress development in the coatings.

In our previous studies [8,13], the effects of single process parameters on specific energy input variation and overlap rate were examined using only one martensitic stainless steel powder, which limited the understanding of how material-dependent properties influence multi-layer cladding behavior. The present work addresses this gap by comparing three different powders (Ferro 55, Castolin 16604, and Metco 41C) under both constant and variable layer-dependent energy input. While Ferro 55 and Castolin 16604 responded favorably to controlled energy variation, Metco 41C consistently exhibited high porosity, low hardness, and tensile residual stresses, confirming that “material–parameter compatibility” was essential for defect-free multi-layer deposition. Overall, this study reveals that layer-dependent energy strategies must be tailored to the specific powder system and cannot be generalized across alloys, providing broader insight into material selection and process optimization for mold repair applications.

## 4. Conclusions

This study examined how powder type and layer-dependent energy input influence the macrostructure, porosity, microhardness, residual stress, and thermal history of multi-layer laser cladding on FGS600-3A ductile cast iron. An experimental approach was employed to evaluate the deposition behavior of Ferro 55, Castolin 16604, and Metco 41C powders.

The results showed that powder composition and thermal properties strongly affected layer quality. Under identical energy input, Ferro 55 produced the lowest porosity and the most favorable hardness profile, while Metco 41C exhibited high porosity, low hardness, and transverse cracking. Castolin 16604 demonstrated intermediate performance, with relatively deeper hardening. The influence of layer-dependent energy input depended on the powder type. For Ferro 55 and Castolin 16604, the application of variable energy input reduced porosity. These results indicate that the effect of variable energy input is not uniform across all materials and should therefore be optimized based on the characteristics of the coating material.

Applying variable energy inputs across layers improved coating quality by reducing porosity and modifying hardness penetration for Ferro 55 and Castolin 16604, although excessively low energy in the outer layers reduced the depth of high-hardness regions. Thermal assessment confirmed that heat accumulation during successive layer deposition influenced the thermal history and contributed to the observed differences in coating properties, particularly for Ferro 55. Residual stress analysis revealed distinct behaviors: Ferro 55 experienced low stresses, Castolin 16604 developed beneficial compressive stresses, and Metco 41C generated high tensile stresses (~490 MPa), consistent with its cracking tendency.

Ferro 55 and Castolin 16604 were identified as suitable materials for multi-layer repair applications when combined with optimized energy input strategies, whereas Metco 41C was limited by its high porosity, low hardness, and tensile residual stresses. These findings demonstrate that the interaction between powder characteristics and layer-dependent energy input governs the resulting coating properties, emphasizing the need for material-specific process optimization.

## Figures and Tables

**Table 1:** Chemical compositions of used materials.

Materials	Fe	C	Si	Mn	P	S	Cr	Mo	Ni	Sn	Cu	Mg	Co
FGS600-3A	Bal.	3.06	2.06	0.52	0.044	0.009	--	--	--	<0.01	0.68	0.049	--
EuTroloy 16604	Bal.	0.20	--	--	--	--	15	2.5	--	--	--	--	15
Ferro 55	Bal.	0.35	0.3	1.1	--	--	7	2.2	7	--	--	--	--
Metco 41C	Bal.	0.03	2.3	--	--	--	17	2.5	12	--	--	--	--

**Table 2:** Process parameters for all specimens.

Sample	Material	Layer	Laser Power (kW)	Scanning Speed (mm/s)	Hatch Space (mm)	Energy Input (J/${mm}^{3}$)	Note
1	Ferro 55	1-2-3	1.3	8	1.05	51.59	Constant parameters
2	Metco 41C	1-2-3	1.3	8	1.05	51.59	Constant parameters
3	Castolin 16604	1-2-3	1.3	8	1.05	51.59	Constant parameters
4	Castolin 16604	1	1.1	12	1.05	29.10	Multi-layer, variable energy input
		2	1.3	8	1.05	51.59	
		3	1.1	12	1.05	29.10	
5	Ferro 55	1	1.1	12	1.05	29.10	Multi-layer, variable energy input
		2	1.3	8	1.05	51.59	
		3	1.1	12	1.05	29.10	

**Table 3:** Results of digital image processing for pore fraction.

Specimen No.	Cross-Section	Pore Area (mm^2^)	Total Area (mm^2^)	Coating Height (mm)	Porosity Ratio (%)	Penetration Depth (µm)	Dilution Rate (%)
1	Transverse	2.55	155.33	5.18	1.64	500	8.81
1	Longitudinal	1.55	70.78	2.36	2.19	400	14.50
2	Transverse	2.36	118.52	3.95	1.99	700	15.05
2	Longitudinal	2.00	72.61	2.42	2.75	700	22.43
3	Transverse	5.14	141.92	4.73	3.62	500	9.56
3	Longitudinal	1.00	95.04	3.17	1.05	500	13.63
4	Transverse	1.76	113.32	3.78	1.55	500	11.69
4	Longitudinal	1.98	66.46	2.22	2.98	400	15.29
5	Transverse	1.26	102.55	3.42	1.23	400	10.48
5	Longitudinal	1.34	81.09	2.70	1.65	400	12.89

**Table 4:** Residual stress values measured by XRD.

Sample	0°	90°	45°	Maximum Principal Stresses (MPa)	Minimum Principal Stresses (MPa)
Sample 1 Ferro 55	−19.6	−90	−9.3	2.7	−112.3
Sample 2 Metco 41	175.8	394.9	456.9	488.9	81.7
Sample 3 Castolin	−261	175.9	−21.2	176.9	−262

**Table 5:** Peak temperatures extracted from thermal histories for each deposited layer.

	Maximum Temperature (°C)			
Sample	Material	1st Layer	2nd Layer	3rd Layer
1	Ferro 55	1628	1375	1750
2	Metco 41C	1623	1758	1654
3	Castolin 16604	1595	1612	1554
4	Castolin 16604	1618	1625	1762
5	Ferro 55	1678	1407	1850

## Data Availability

The data supporting the discussion in this study are available in published reports and can be provided upon request.
